# Experiences of current vital signs monitoring practices and views of wearable monitoring: A qualitative study in patients and nurses

**DOI:** 10.1111/jan.15055

**Published:** 2021-10-15

**Authors:** Carlos Areia, Elizabeth King, Jody Ede, Louise Young, Lionel Tarassenko, Peter Watkinson, Sarah Vollam

**Affiliations:** ^1^ Critical Care Research Group Nuffield Department of Clinical Neurosciences University of Oxford Oxford UK; ^2^ National Institute for Health Research Oxford Biomedical Research Centre Oxford UK; ^3^ Therapies Clinical Service Unit Oxford University Hospitals NHS Foundation Trust Oxford UK; ^4^ Department of Engineering Science Institute of Biomedical Engineering University of Oxford Oxford UK; ^5^ Kadoorie Centre for Critical Care Research and Education Oxford University Hospitals NHS Trust Oxford UK; ^6^ Present address: Kadoorie Research Centre Level 3 John Radcliffe Hospital Headington Oxford OX3 9DU UK

**Keywords:** acute care, acute nursing, alarms, clinical data, deterioration, escalation, monitoring, nursing, vital signs, wearables

## Abstract

**Aims:**

To understand current experiences of vital signs monitoring of patients and clinical staff on a surgical ward, and views on the introduction of wearable ambulatory monitoring into the general ward environment.

**Design:**

Qualitative study.

**Methods:**

Semi‐structured interviews using topic guides were conducted with 15 patients and 15 nurses on a surgical ward between July 2018 and August 2019. The concept of ambulatory wearable devices for clinical monitoring was introduced at the end of the interview.

**Results:**

Three interconnected themes were identified. Vital sign data as evidence for escalation, examined nurses' use of data to support escalation of care and the implications for patients perceived to be deteriorating who have not reached the threshold for escalation. The second theme, Trustworthiness of vital sign data, described nurses’ practice of using manual measurements to recheck or confirm automated vital signs readings when concerned. The final theme, finding a balance between continuous and intermittent monitoring, both patients and nurses agreed that although continuous monitoring may improve safety and reassurance, these needed to be balanced with multiple limitations. Factors to be considered included noise pollution, comfort, and impact on patient mobility and independence. Introduction of the concept of ambulatory wearable devices was viewed positively by both groups as offering solutions to some of the issues identified with traditional monitoring. However, most agreed that this would not be suitable for all patients and should not replace direct nurse/patient contact.

**Conclusion:**

Both patients and staff identified the benefits of continuous monitoring to improve patient safety but, due to limitations, use should be carefully considered and patient‐centred.

**Impact:**

Feedback from nurses and patients suggests there is scope for ambulatory monitoring systems to be integrated into the hospital environment; however, both groups emphasized these should not add more noise to the ward nor replace direct nursing contact.

## 1 INTRODUCTION

Failure to monitor patients is associated with adverse outcomes and delayed recognition of deterioration (National Institute for Health and Care Excellence (UK), 2020). In hospitals, routine monitoring practice involves vital signs measurements (oxygen saturation, temperature, blood pressure, heart rate and respiratory rate) and Early Warning Scoring (EWS) systems. These observations can be taken intermittently at a protocolized frequency, or in the presence of acute deteriorations can also be measured through the use of hospital monitors which continuously capture vital sign data (Hravnak et al., 2008). These monitors are usually wired and static, meaning patients’ movements are restricted during use (Bonnici et al., 2012).

### 1.1 Background

Intermittent vital sign measurement is the most commonly used method of clinical monitoring on surgical wards in the UK (Cardona‐Morrell, Prgomet, Lake, et al., 2016). Aggregate scores from measurements contribute to the early warning scoring (EWS) systems, highlighting physiological vital signs abnormalities (Royal College of Physicians, 2017) contributing to the recognition and escalation of deteriorating patients. However, several factors can impact monitoring frequency, such as clinical shift duration (Dall'Ora et al., 2019), ward staff levels (Griffiths et al., 2018; Redfern et al., 2019) and vital sign measurements related workload (Dall'Ora et al., 2020), meaning the ideal frequency is often not achieved (Jansen & Cuthbertson, 2010a) with only partial adherence to the vital signs monitoring protocol (Hands et al., 2013), especially at night (Hope et al., 2018). Consequently, it is recognized that incomplete sets of observations can lead to further delays in deterioration recognition (Cardona‐Morrell et al., 2016).

Continuous monitoring is an alternative to intermittent measurements and is seen by staff as a positive tool to support early deterioration identification, as vital signs are being monitored all of the time and deterioration of a patient's condition should be easier to identify, provided that there are no false alerts (Downey et al., 2018). Previous research suggests it may be feasible to implement continuous monitoring outside intensive care units (ICU) with the potential to improve patient safety, despite its cost‐effectiveness inside general wards not being well established (Downey, Chapman, et al., 2018; Javanbakht et al., 2020). Previous evidence suggests that current continuous monitoring systems might also pose a barrier to patient mobility and independence, affecting its adherence in the less acute population (Bonnici et al., 2012; Downey, Chapman, et al., 2018).

Wearable technology, or “wearables”, are electronic devices that can be worn as accessories by the user. Wearable vital sign monitoring technology is growing fast and progressively influencing healthcare (Soon et al., 2020; Weenk et al., 2020) as it can potentially facilitate continuous monitoring in the hospital environment (Michard et al., 2020; Santos et al., 2021; Weenk et al., 2017a). As these tend to be wireless, they offer an increase in comfort and can be less restrictive than traditional monitoring while supporting clinical staff by providing regular vital signs data (Leenen et al., 2020; Sun et al., 2020; Weenk et al., 2017b). However, research suggests that introducing these devices to the hospital environment may have physical or psychological effects that should be considered (Downey et al., 2018).

## 2 THE STUDY

This study is one of the first phases of the Virtual High Dependency Unit project, a collaboration between the Institute of Biomedical Engineering and clinicians from the Nuffield Department of Clinical Neurosciences at the University of Oxford. This project aims to refine and integrate ambulatory monitoring systems for use in clinical practice.

### 2.1 Aims

The primary objective of this qualitative study was to understand patients' and clinical staffs' current perceptions of current monitoring practices. We also sought participants' views on the feasibility of using wearable ambulatory continuous monitoring in the hospital environment.

### 2.2 Design

Qualitative study based on one‐to‐one interviews of nurses and patients in a surgical ward.

### 2.3 Participants

During the qualitative interviews, maximum variation sampling was used to engage with a range of staff (band level (NHS Employers, 2021) and nursing years) and patients (age and length of stay) to access a range of experiences (Baker & Edwards, 2012; Mason, 2010). Nurses and patients were interviewed as part of a service evaluation of current monitoring practices on a surgical ward at a large teaching hospital in the UK. We aimed to recruit up to 15 participants from each group, or until saturation of data was achieved, based on the relatively narrow aim of this service evaluation to explore perceptions of monitoring use (Baker & Edwards, 2012; Mason, 2018; Mason, 2010). Although data saturation is not aligned to thematic analysis, we aimed to achieve this as an indication of reaching a sufficient sample.

### 2.4 Data collection

The interviewers encouraged the interviewee to talk freely about their experiences (Pope et al., 2000), using a topic guide to direct follow‐up questions and prompts where needed. Although the interviewers had an agenda of topics to be discussed (Appendix 1) this interview format allowed interviewees to diverge and guide any subsequent questions. We also introduced the concept of ambulatory monitoring at the end of each interview, as part of the aim of this service evaluation was to inform the development of an ambulatory monitoring system.

Participants were aware that interviewers were not part of their nursing team and this study would not impact their care and were informed of the reason and outcomes of this study before consenting to be interviewed. Clinical staff was also made aware that the interviews were not part of their employment and would not have an impact on their job; they were informed on the objectives of the study and reason for their participation before consenting. All interviews were conducted in a quiet area of the ward, audio‐recorded and lasted up to 30 min. Recordings were professionally transcribed and accuracy checked by the research team before analysis.

### 2.5 Ethical considerations

This service evaluation is part of our virtual high dependency unit project (vHDU), with the overall aim of testing the feasibility of deploying ambulatory vital sign monitoring in the hospital environment. This study was approved as a service evaluation of monitoring practices in a surgical ward inside the Oxford University Hospitals NHS Foundation Trust (Clinical staff interviews DATIX ID:5168 and patient interviews DATIX ID:5491). All participants were approached in the surgical ward and informed of the aim of the research. Consent to participate was taken and anonymity and privacy were guaranteed through the use of study numbers and anonymization of transcripts. Study participation was voluntary and there was no conflict of interest between participants and researchers.

### 2.6 Data analysis

Interviews were analysed using thematic analysis (Braun & Clarke, 2013; Guest et al., 2012) and NVivo 12 (NVivo qualitative data analysis software; QSR International Pty Ltd. Version 12, 2018). We selected a thematic analysis approach as this is well aligned to the pragmatic aims of the wider mixed methods study which this work is part of (Bowers et al., 2013; Creswell & Clark, 2017; Teddlie & Tashakkori, 2009). Data saturation was confirmed through team discussion of transcripts as they were available, with an agreement that no new themes were being identified.

Thematic analysis followed the six phases described by Braun and Clark (Braun & Clarke, 2006). Analysis was initiated after the first interviews were concluded and continued alongside data collection. In the first phase, familiarization with the data, transcripts were read through several times by two researchers. Proceeding to phase two, initial codes were assigned to portions of the text and developed through comparison and discussion. Codes were further developed through discussion with a third reviewer, during which codes were grouped into coding trees using both NVivo and paper‐based approaches (phase three). Further coding was undertaken by the same two researchers, using this coding tree (phase four). Theme development was undertaken through the use of mind maps and team discussion until the final theme structure was agreed (phase five). Themes were interlinked and these relationships are identified throughout.

### 2.7 Rigour

This manuscript follows the Consolidated criteria for reporting qualitative research (COREQ) guidelines (Tong et al., 2007). All interviews were conducted by two female registered nurses, both trained in qualitative research, one is MSc holder. Neither of the interviewers had any previously established relationships with participants (both nursing staff and patients). Pre‐assumptions or bias concerning the research questions were discussed within the research team throughout data collection and analysis. Researchers also provided a reflective account of each interview as part of the case report form, including observations on rapport, the setting of the interview, and any potential biases. The team approach to analysing and developing themes also contributed to reducing bias.

## 3 FINDINGS

A purposive sample of thirty participants (15 clinical nurses and 15 patients) were interviewed between July 2018 and August 2019. None of the approached nurses and patients refused to participate. The mix of gender, range of bands and nursing experience were broadly reflective of the ward nursing workforce. Group's demographics are outlined in Tables 1 and 2. We interviewed six female and nine male patients, age ranged from 30 to 91 years old (median 68), also reflective of the ward population.

**TABLE 1:** Clinical staff interviewees demographic details

Clinical staff demographics			
Interview number	Sex	Nurse band^a^	Years qualified
N1	Female	5	<1
N2	Female	5	2
N3	Female	5	3
N4	Male	5	2
N5	Female	5	<1
N6	Female	7	9
N7	Female	6	9
N8	Female	6	2
N9	Female	6	5
N10	Female	7	6
N11	Female	6	7
N12	Male	5	5
N13	Male	6	7
N14	Female	5	5
N15	Female	6	>10

^a^ Nursing band level refers to the UK National Health Service (NHS) Agenda for Change (NHS Employers, 2021), categorising roles and experience within these band levels, with defined salary levels and increments, making it easier to move between NHS organisations.

**TABLE 2:** Patient interviewees demographic details

Patient demographics		
Interview number	Sex	Hospital LOS
P1	Female	22
P2	Male	112
P3	Female	21
P4	Male	17
P5	Female	28
P6	Female	28
P7	Male	18
P8	Female	39
P9	Male	6
P10	Male	21
P11	Male	4
P12	Male	1
P13	Female	14
P14	Male	28
P15	Male	30

Abbreviation: LOS: Length of stay.

In this paper, we present three main themes identified through thematic analysis (Table 3). The first theme, *Vital sign data as evidence for escalation*, explores how nurses described using vital sign data to inform and defend their decision to escalate. In the second theme, *Trustworthiness of vital sign data*, the importance of trust in data is discussed, how nurses responded to concern about accuracy of vital signs readings. The final theme, *finding a balance between continuous and intermittent monitoring*, examined the decision‐making underlying monitoring selection, and included three sub‐themes: *reduced staff contact versus patient safety*, *negative aspects of continuous monitoring and individualized patient monitoring*. At the end of each interview, we introduced the concept of ambulatory monitoring, and report the findings of this in the final section: *ambulatory wearable devices for clinical monitoring*.

**TABLE 3:** Theme map

Theme One: *Vital sign data as evidence for escalation*
Nurses use vital sign data to support their concern and facilitate escalation, which may be challenged when patients do not reach the threshold for action but staff are worried about them. Where this occurred, nurses were willing to risk criticism from colleagues our ensuring patient safety.
Theme Two: *Trustworthiness of vital sign data*
Trustworthiness of data was identified as a key consideration in monitoring of vital signs. When nurses were worried about accuracy, they described instinctively returning to ‘manual’ measurements to ensure the data were reliable.
Theme Three: *Finding a balance between continuous and intermittent monitoring*
In this theme, three sub‐themes are explored. There are both benefits and limitations to continuous monitoring, including increased patient safety and reassurance, but reduced comfort and mobility. These aspects require careful balancing in the context of individualized patient care.

### 3.1 Vital sign data as evidence for escalation

Nurses commonly described vital sign and early warning score data as providing hard evidence for escalation, in essence quantifying that a patient's clinical status has changed. Some staff described initial distrust of the score but acknowledged that in particular, this might guide more junior staff to recognize when a patient was deteriorating. This indicated that more experienced staff may use clinical judgement beyond the data included in the early warning scoring system.

'… when it first came out I have to admit I didn't really think it was necessary because I come from before track and trigger but … I think it's really good especially for the newbies they find it really helpful just put their thoughts in order really and you can't go wrong red is bad green is good' (Nurse 15).

When a patient seemed to be deteriorating but was not reaching the threshold for escalation (also referred to as 'triggering' throughout the interviews), nurses described gathering and presenting clinical data, including vital signs, to doctors to support their worry and, when appropriate, the need for escalation. Nurses reported it was easier to recognize deteriorations if they had nursed the patient previously as it allowed them to identify changes in their condition from a “normal” baseline.

'… like he's not right and umm then they don't take you seriously and they're like yeah but his early warning score is zero he's fine, he's fine or whatever and it's like no I'm telling you and then obviously the patient deteriorates and you are like actually, I did try and sort of flag it up earlier that I was concerned about the patient' (Nurse 04)

'Because you get to know how they are normally and how they are so you can recognise quickly if something’s not normal for them and out of character' (Nurse 08)

When escalating a deterioration, staff reported 'knowing their team' enabled trust and more effective communication. This was enhanced if a member of staff had worked there for a considerable time period and could identify the most appropriate clinician to escalate a particular patient/deterioration event. However, the attitude of a staff member may hinder this communication and was often heightened in times of staff shortages and limited bed capacity.

'The attitude of the person on the other end of the phone. Really some people will believe you some people won't simple as that.' (Nurse 15)

'Far better to look a total idiot and report something that doesn't need reporting then have someone arrest because you didn't do anything about it' (Nurse 15).

In summary, nurses described how they use vital sign data to support their concern and facilitate escalation. They also described current challenges and delays during escalation, especially when a patient is not triggering. However, most agreed that it is always better to escalate, with nurses describing their willingness to be criticized by their colleagues for escalating unnecessarily rather than risk the safety of a patient.

### 3.2 Trustworthiness of vital sign data

Trust in continuous monitoring was reported as varying depending on patient status and perceived device accuracy. Nurses described using several strategies to investigate and troubleshoot potentially erroneous vital signs readings related to patient condition, such as cold peripheries and clammy skin, which may affect device function.

'…you have to be careful when you’re interpreting that result as well umm because obviously if it’s a finger probe and they are peripherally very cold err that can often not give you a reliable result…' (Nurse 7),

'the first thing you do is look at the chest and make sure that the stickers on properly because they fall off all the time. Someone's unwell they're cold, they're clammy their skins all sweaty and horrible, the things won't stick so you know it's just a matter of using your nous [common sense] really to sit through and work out what's physiological and needs worrying about and what’s mechanical and could be ignored.' (Nurse 15)

Many nurses identified variable reliability between methods of vital signs measurement. For example, some nurses perceived measurement of pulse rate as more reliable if using a cardiac monitor (3‐Lead electrocardiogram ‐ ECG) or manual checks compared with a pulse oximeter, even though this was the more commonly used method for recording pulse rate. Manual assessment of pulse rate was described as offering more information than automated devices.

'… if you’ve got someone on 3 lead monitoring I would say the heart rate on that is pretty reliable if it's a cardiac monitor (Nurse 7).

'We take manual pulses as there is a lot, you know, the monitor can't tell you about the feel of it, if they have got a bounding pulse or if it is thready [weak and rapid] or if it, there are other pieces of information that you gain by doing manual observations' (Nurse 6),

'if I wasn't sure if I, say for example, if the heart rate said 155 I would do it manually [to] double‐check' (Nurse 11),

'Manual' checking was also commonly advocated for blood pressure measurements, with nurses describing often checking manual blood pressure readings with a sphygmomanometer and stethoscope when automated results were out of normal range.

'I find the blood pressure if their extremely hypo/hypertensive, the machine is not as accurate as a manual blood pressure reading' (Nurse 7).

'… if the blood pressure was lower then I would do a manual blood pressure or manually check their heart rate. Instead of just relying on the Dynamap [automated blood pressure monitor].' (Nurse 14)

Concerns around the reliability of automated readings were even more pronounced for respiratory rate, and most nurses described assessing respiratory rate manually as those derived from ECG monitoring were deemed insufficiently reliable.

'… because the monitors that we have are notoriously poor at picking up things like respiration rate which I wouldn't always rely on those monitors' (Nurse 10)

'… if the resps [respiratory rate] said 8 I would do them manually to make sure that what I was getting off the machine was correct' (Nurse 11)

Oxygen saturations were acknowledged to be variable as well and nurses described using different probes and devices to double‐check measurements. There was no reference to temperature measurement accuracy or trustworthiness, perhaps due to the lack of alternative means of measurement.

'… oxygen sat[uration]s I can say can be quite variable as well'(Nurse 7),

'I had a patient that on the monitor even with ear probe umm it said that the [oxygen] sat[uration]s were 88% for example and I’d put them on the normal obs[ervation] machine just to double‐check that those sats were correct and the normal obs machine said 97%' (Nurse 11).

In summary, the majority of the interviewed nurses discussed the importance of trustworthiness of vital signs data. When they were worried about accuracy, they described instinctively returning to ‘manual’ measurements to ensure the data were reliable.

'I think if you know if you are concerned or it’s something acute that’s changed it’s always better to check those things manually anyway when you’re going through your ABC approach and you’re reviewing them.' (Nurse 7).

### 3.3 Finding a balance between continuous and intermittent monitoring

Three sub‐themes contributed to this overall theme. The first sub‐theme, reduced staff contact versus patient safety, explores the impact on staff contact from continuous monitoring, which was balanced with the perception of increased patient safety during busy periods when nurses were unable to return to the bedside to measure vital signs. In the second sub‐theme, two key identified drawbacks of continuous monitoring—restricted mobility and alarm noise—are discussed. In the final sub‐theme, individualized patient monitoring, the process of balancing these considerations is explored.

#### 3.3.1 Reduced staff contact versus patient safety

Patients reported mixed views regarding continuous monitoring. Some indicated it provided them and their relatives with reassurance that the alarms could attract the attention of staff to attend their loved one. However, some nurses highlighted that continuous monitoring could also sometimes cause distress by making patients aware of their condition. In contrast, some patients recounted becoming worried when they recognized their vital sign values were abnormal but were reassured this was picked up by clinical staff.

'… yes I think it gave a sort of sense of a feeling of security a bit, being looked after.’ (Patient 8).

'Although we do try and reassure them that it’s because we are taking very good care of them and, you know, we want to see any signs of anything we can sort out. I think for patients it can seem quite a big deal and it tells them that perhaps they are sicker perhaps than they thought they were.' (Nurse 6).

'It worries me more if my blood pressure is low because it has been a couple of times but I know that I'm in the right place if anything’s happening anyway and they're going to monitor and deal with it and so I’m not scared if you know what I mean.' (Patient 7)

Furthermore, a concern often raised in relation to continuous monitoring was the impact this may have on reducing interactions between patients and nurses. Several patients described the manual vital sign measurement as a comforting interaction and a chance to talk to staff, providing an opportunity to discuss their condition and voice their needs.

'The nurse one [manual observations] is good. You've got a human touch and someone comes to speak to you and that's good but what's bad about it as they might not come quite so regularly… I don't like to use it all the time the call bell and so yes I'd wait if it wasn't terrible.' (Patient 9)

'… and I think it’s nice to have that regular nurse contact coming round. I mean they deal with the drugs and the medications as well that’s true but it’s a nice opportunity to talk to people… it was quite nice for me as well because I was quite light headed and found my blood pressure was down and that was an explanation for it and so that was quite good.' (Patient 1).

Nursing staff also valued this contact, which some described as offering a valuable insight into a patient's status, as they could visually observe the patient and often recognize signs and symptoms of non‐triggering deteriorations.

'I believe intuition is a great thing as well so experience and intuition. And you’ll be like this patient's not right. Although they could be triggering maybe a zero but you just know in you, you know because you know the patient, you just know there’s just something not right the patient could deteriorate after that' (Nurse 4)

In contrast, several nurses highlighted the benefits of continuous monitoring to patient safety, as they could glance at the monitors to easily review a patient's status. This suggests continuous monitoring improves confidence in managing and prioritizing their caseload. Furthermore, there was recognition from some nurses that vital signs may change in the time between intermittent observations (usually 4 hours in stable patients), which could be more quickly identified by continuous monitoring.

'It does feel safer, that's what I would say. And I just feel like, sort of that I am doing a better job, like, if I dunno, if you are picking up things quicker, you can solve anything that can go wrong quicker and you are more efficient. Um, if an alarm is going off when you are not there and the doctor sees it, then I feel like, it's their moral code they would do something (laughs) you would hope.' (Nurse 2).

'I guess you're more likely to pick up on things that are changing quite slowly if someone's blood pressure drops a little bit and you're doing it more thoroughly and more frequently you'd be able to act on that sooner rather than if you left it four hours and then saw a massive drop and you could intercept and do something about it.' (Nurse 9).

#### 3.3.2 Negative aspects of continuous monitoring

A significant drawback of continuous monitoring, identified by both patients and staff, was the impact on mobility. Several patients mentioned that the continuous monitoring can sometimes be uncomfortable and impair mobilization due to the added challenge (e.g., wires) and perceived condition severity; resulting in being largely limited to the bed or bedside.

'you just end up going from bed to chair don't you' (Patient 2),

'I tried to move in the bed and was tied to everything everywhere' (Patient 6).

Nursing staff also identified this limitation in interviews. Additionally, most nursing staff showed increased concern for confused patients, as the monitoring wires have the potential to increase the risk of falls, which was also echoed by one patient.

'They feel like they can't move with it on. Not that they want to lie there, it just makes it harder for them to mobilise and do things for themselves so, in some instances it does actually increase our workload … it kind of ties the patient down and it affects other areas of their care' (Nurse 2).

'Well you can't move because you've also got a load of bags hanging on to you from where everything's being drained and so you can't really move about (a) because you'd probably trip over but also you’ve got all this other kit to carry plus where you've been in bed so long your muscles are wasting away and you've just got no strength.' (Patient 7).

The noise from continuous monitoring alarms was also raised as a concern by both patients and nursing staff and was linked to frustration and anxiety. For patients, this frustration related to the actual noise, either from their alarm or their neighbours and worry about why the alarm was sounding'. Some nurses also mentioned this distress to be augmented by the continuous monitoring alarms that can be disconcerting for both patients' relatives due to the perception that their loved one is deteriorating.

'I suppose if the machines keep bleeping and things like that if you don’t know what they’re for that can be a bit disconcerting and the alarms going off for no reason’ (Patient 7).

'I think it is very disconcerting for relatives. Apart from anything else,, as the nurses and doctors know they alarm frequently. If they lose the signal, because somebody has moved, or something like that, or the parameters haven't been set up in a certain way, then it will alarm constantly. And I think that relative perception of those alarms going off is that their relative is, is deteriorating and I think it can be quite panicking for them to hear beep beep beep when their relative is on the monitor.' (Nurse 6)

Some patients suggested that the constant background noise may lead to alarm fatigue from the nurses, resulting in lack of action upon the alarms. Several nurses discussed adjusting the alarm settings to reduce noise and minimize false alarms.

'… they [clinical staff] really don't hear them I don't think anymore' (Patient 1),

'you can hear them in the background all the time and you get used to them.' (Patient 5)

'Well for example you have some patients who are known as COPD and they don't need to have saturations of oxygen at around 94% so we normally set those alarms according to the patients' normal rates. For a patient who's got AF obviously and we know he's got AF as a basic we disconnect the alarm of the AF detector because it's just going to jump all the time and things like that.' (Nurse 13).

However, nurses raised safety concerns related to changing alarm thresholds, with some suggesting they minimized adjusting these or sought guidance from a doctor before making a change in patients alarm thresholds. Concerns were also raised related to patient safety if alarms were not returned to their baseline settings before being attached to the next patient.

'…and what you would do, is in collaboration with the surgeon is find a range for target saturations and they might not be the 95‐100% that most people are. They might have a totally different set of parameters 88‐92 for example.'(Nurse 6)

'… if you start fiddling around with the alarms you might change the alarms for this patient and not change them back when you've taken that monitoring off so the next patient that comes along may be sick and you haven't noticed it because the alarm hasn't gone off because it's been reset' (Nurse 15).

#### 3.3.3 Individualized patient monitoring

Most nursing staff discussed the benefits of continuous monitoring during busy periods, suggesting this may save them time while increasing efficiency in prioritizing patients, identifying trends and detecting deteriorations. This was suggested to contribute to the decision‐making process of continuously monitoring one or more patients.

'Just because when you are busy doing other things you can make sure that, just from a glance that nothing has changed. That is the main thing. Especially with post‐op patients, and you have got a really poorly pancreatitis patients that are acutely ill.' (Nurse 1)

However, most nurses asserted that not all patients require continuous monitoring and this should be prioritized according to patient condition, staff levels and their own clinical judgement. In addition to these considerations, there was also a suggestion from some staff that decisions may be based on monitor availability and prioritization of need as well.

'A patient which is physically fine … doesn't need a constant monitor, Just need to check regularly to make sure that everything is still going fine, … because most of the patients don't need the monitoring, sometimes they are [scoring] a 1 or a 2 it's their normal blood pressure, their normal heart rate which may be a bit higher or lower than normal, so basically you will have to take continuous monitoring for the patients who truly need it' (Nurse 5).

General tolerance to continuous monitoring was identified as variable and linked to a number of factors, including patient condition, severity, independence, and expectations. These factors may contribute to the nurses' decision to continuously monitor a patient. Nurses described several approaches to encouraging patients to wear monitoring, including reassurance, negotiating rest periods and explaining the purpose of the monitoring.

'You tolerate quite a lot when you're unwell actually. You don't mind the noise and everything whereas when you're well its really annoying' (Patient 1).

'They sometimes like it removed. So then you have to compromise, and say “Ok, well we'll take it off for half an hour but I need to pop it back on”. Explain the need behind it and they are usually OK with it.' (Nurse 1),

'Sometimes confused patients don't tolerate it. They don't understand it and they find it annoying and stuff and that's quite hard for them and so try and one‐to‐one them as much as possible to keep them calmer and understand so they don't rip it all off' (Nurse 8).

Summarizing this overarching theme, both patients and nurses reported that while intermittent monitoring can facilitate human interaction and provide an opportunity for building the patient‐nurse connection, continuous monitoring can improve patient safety and support clinical staff during busy periods, promoting efficiency and aiding prioritization. However, not all patients need this type of monitoring and the decision for continuously monitoring needs to be carefully considered, weighing the positive/negative impact on an individual patient basis while considering ward/environment demands. Both groups also discussed the impact of noise pollution created by the continuous monitoring alarms, also acknowledging the alarm fatigue felt by the nursing staff.

### 3.4 Ambulatory wearable devices for clinical monitoring

The concept and potential for ambulatory monitoring were introduced to each interviewee. Initial responses from nurses were generally positive and linked back to the earlier discussion of weighing the benefits of closely monitoring patients with the physical limitations of traditional wired systems.

'I think, yeah I guess there would be a benefit of patients having ambulatory style monitoring … some of the elderly patients with dementia that you want to monitor them and you need to monitor them but they’re up and about and trying to get out of bed all the time and they want to go for walks and whatnot. That would be quite good because they're forever taking their monitor off or you know pulling the [brand name] monitor down the ward with them that would be good.' (Nurse 7)

'That everybody would be monitored a little bit more closely and even if they do look well they do quickly deteriorate' (Nurse 8)

However, some of the nurses voiced concern that it would further reduce the frequency of manual observations and direct patient contact, identified in earlier themes as an important opportunity to offer reassurance and visually assess patients. One patient emphasized this by suggesting an ambulatory monitoring system may benefit staff more than patients, with a resultant reduction in staff contact.

'Just because it would make people quite lazy if they think, if they aren't motivated to do the job then I think it would be a way that people could be quite lazy and just be like “Yeah they are fine” But yeah, I dunno' (Nurse 2).

'I should imagine it wouldn't be that much good for us, it would be more for the nurses so they would stop coming round every minute and they could just see it I suppose on the computer and what's going on.' (Patient 4)

In contrast, some patients also recognized the potential of ambulatory monitoring systems to support nurses to use their time more efficiently. Most patients also suggested they would be more independent and able to mobilize if continuous monitoring was less invasive, a key limitation discussed in previous themes. Most patients saw ambulatory monitoring systems as a positive addition to their care.

'Well yes, I think it must take some of the grinds off of them. If they can just walk up, look at his watch or whatever it is and put that down and that is accurate. Accuracy is the thing' (Patient 15).

'It probably would yes because you can also be monitored while you're moving about and get the heart rate when you're exercising and things like that.' (Patient 7), 'Well is just less obtrusive than this and if you were more mobile, I'm not at the moment but if I were more mobile I could walk off to the toilet and not worry about it.' (Patient 8).

All nurses interviewed also agreed this system should alert them of clinical deteriorations. However, there were suggestions these alerts should not add more noise to the ward environment and should instead focus on other types of stimulus that would capture clinicians' attention. In discussing the potential for alerts nurses made several suggestions to avoid adding additional noise to the ward while still engaging them to see the patient. These included staff‐held devices that vibrated or flashed to alert nurses to a change in a patient's condition.

'…if we all had [handheld devices] or something in our, you know like how we all carry phones around basically, like something that could on like an [handheld device] that was really simple that had all your patients for the day like the SEND [electronic vital signs] system, then you, it would like to vibrate if, I dunno someone was, something was happening' (Nurse 2)

'Well, I am not a fan of alarms, but at least if you hear an alarm you don't have to be looking at anything in particular. Um, I am just thinking I don't know a flashing light, I suppose the problems with areas I say that if you are not looking at it at the time. Or perhaps I am just thinking out loud, maybe like a pager or something that you carry that vibrates.' (Nurse 6)

In summary, ambulatory monitoring systems were seen as a potential facilitator for early deterioration detection, while promoting patient mobility and independence. However, it should not add more sound alerts into already 'noisy' environments and nurses provided other alternatives that should be explored. In the end, both nurses and patients agreed that this technology should support staff in their clinical decision‐making, and not replace it.

'…you will always need someone behind that technology, a qualified nurse to understand all the signs and symptoms and who can interpret all those numbers. It would be helpful but I don't think it's a cure for everything. You need someone with experience or someone who understands what they’re doing to read all those numbers.' (Nurse 13)

## 4 DISCUSSION

This study explored current monitoring practices and experiences by patients and clinical staff working in a surgical ward. Three main themes were generated during interviews. A fourth topic was also introduced to understand staff and patients views on *wearable ambulatory monitoring devices* and its implementation in the hospital environment.

*Vital sign data as evidence for escalation* was a theme identified by the nurses when discussing current experiences and challenges in deterioration detection and escalation. Familiarity with a patient's presentation and baseline status enabled nurses to readily recognize perhaps subtle or marked changes in clinical conditions that would trigger their attention. There was good awareness of how to escalate a patient based on the local early warning score (EWS) process as nurses reported harnessing available vital sign and EWS data when communicating with doctors. One of the most commonly reported challenges seemed to be supporting their worry when a deteriorating patient was not triggering. This concern is not new (Prgomet et al., 2016) and is also explored as the nurse‐worry‐factor (Odell et al., 2009). Interestingly, evidence supports this sense of worry by the nurses, sometimes even outperforming some EWS on patient deterioration detection (Romero‐Brufau et al., 2019). However, in our study, most nurses reported that it was challenging to justify the need for patient escalation without any data to evidence concerns (Ede et al., 2020).

*Trustworthiness of vital sign data* was the second identified theme, where nurses shared their views on accuracy of readings, and most agreed to commonly double‐check heart rate, respiratory rate and blood pressure measurements manually when in doubt of the automated measurements reliability (Prgomet et al., 2016). This is also experienced in oxygen saturations when the ear probe is unreliable or the finger probe is not well placed; no concerns were shared regarding temperature measurements. Nurses reported automated respiratory rate from the continuous monitor (derived from 3 Lead‐ECG) to be the most unreliable and clinical staff tended to reject its measurements. This has previously been reported in the literature (Prgomet et al., 2016)e and we believe it to be an important finding. Although there are studies validating the different methods of vital sign monitoring, little is known about the trustworthiness of each method, as perceived by the clinical staff, despite these methods being part of their daily practice.

The theme *finding a balance between continuous and intermittent monitoring* included three sub‐themes. In the first, *Reduced staff contact versus patient safety*, patients reinforced the importance of nursing contact and how manual vital sign observations create an opportunity for human interaction; as previously described in other studies (Downey, Brown, et al., 2018). Nurses also emphasized the importance of face‐to‐face contact to enable visual assessment of the patient and found this to be a valuable opportunity to detect clinical changes that may not contribute to the EWS data. Similar to previous evidence, some participants described staff shortages and ward demands as contributing to suboptimal manual monitoring frequency and potentially delaying deterioration detection (Clifton et al., 2015; Dall'Ora et al., 2019; 2020; Downey et al., 2017; Griffiths et al., 2018; Jansen & Cuthbertson, 2010b). In this instance they proposed that continuous monitoring could be beneficial both at a patient and ward level, promoting safety and efficiency (Prgomet et al., 2016). However in the second sub‐theme, *negative aspects of continuous monitoring*, most nurses reinforced that not all patients require continuous monitoring and the decision for the type of monitoring encompassed not only individual patient needs but also the ward environment. The noise pollution created by these monitors was also discussed, as patients commonly found the noise from the alarms to be frustrating and distressing, often resulting in sleep disruptions. Conversely, patients felt nurses subconsciously disregard the alarms from the noise pollution and developed alarm fatigue, a known concept previously described in other studies (Bonafide et al., 2015; Drew et al., 2014; Görges et al., 2009; de Man et al., 2013). Some nurses recognized that alert thresholds can be adjusted to reduce false alarm rates; however, in some cases, this is previously agreed with a doctor. In the last sub‐theme, *Individualized patient monitoring*, both groups provided mixed views on this as while continuous monitoring was perceived to improve patient safety and reassurance while supporting staff in managing their caseload and prioritizing other activities/patients, it can also be restrictive, reduce patient independence and cause distress (Prgomet et al., 2016). Considering this, tolerance to continuous monitoring was reported to be variable and also contributed to the clinical decision of its use. In this theme, we have highlighted the complexity of developing an ambulatory monitoring system that balances the need for increased surveillance of vital signs with patient comfort and reassurance. Future developments in this field will need to accommodate both patient safety and comfort if they are to be successfully implemented into clinical practice (Barr et al., 2021; May et al., 2016).

*Ambulatory wearable devices for clinical monitoring* was also introduced at the end of the interviews to explore perceptions of how this may influence monitoring practices. Both nurses and patients saw the benefits of ambulatory monitoring systems and welcomed this type of monitoring to allow for increased mobility and improved tolerance while promoting early deterioration detection through ambulatory continuous monitoring. However, nurses identified the need for careful consideration of integration of this data into existing hospital systems and the alerting of deteriorations within the technology. In particular, they were keen to avoid adding to the noise pollution suggesting other stimuli such as vibration or visual cues, and for these devices to be user friendly within the clinical environment. However, both interviewee groups agreed that this technology should support clinical staff, and not replace it (Prgomet et al., 2016). As the deployment of wearable technology in hospital environments is still mostly in the feasibility stage (Leenen et al., 2020), clinical feedback is crucial for its successful implementation in these environments, if its potential for enabling early deterioration detection is to be realized (Leenen et al., 2020; Sun et al., 2020).

## 5 LIMITATIONS

This study is not without limitations. First, we recruited a relatively small sample size of patients and nurses. However, given the relatively narrow focus of the study, purposive sampling and richness of the data generated, the sample size was considered sufficient to fulfil our study objectives. Furthermore, within the team we agreed that data saturation had been achieved – i.e., no new codes were being generated from new interviews. Including other members of clinical staff (e.g., doctors, healthcare assistants, allied health professionals) may have elicited broader perspectives of monitoring practices, nonetheless, we mainly focused on nursing staff as they are the frontline clinicians in regards to vital sign monitoring and associated clinical decision‐making related to escalation (Smith et al., 2020). Furthermore, we believe our study results are consistent with previous evidence and representative of monitoring experiences in this population.

Second, this study was conducted in a single surgical ward, selected as the site for our future vHDU project (and was part of a wider project assessing device wearability and accuracy Areia, Vollam, et al., 2020; Areia, Young, et al., 2020). The transferability of our results is therefore limited. Additionally, the setting for some of the interviews was also suboptimal, and although we made efforts to provide a friendly and private environment, this was not possible on some occasions, and interviews were conducted at the bedside for some patients, which may have had an impact on their willingness to share their experiences.

## 6 CONCLUSION

There was agreement by patients and nurses that the decision to continuously monitor should be carefully considered and adapted to each situation, as although continuous monitoring may improve safety and reassurance, it can also be disruptive for some patients and, when concerned, nurses tend to double‐check the majority of vital signs manually to ensure reliable measurements. There is a scope for the introduction of ambulatory monitoring systems and it is seen positively by patients and clinical staff to bridge the gap between the traditional wired continuous systems and manual observations; however, both groups reported that these should not add more noise to the ward environment nor replace clinical contact.

## AUTHORS' CONTRIBUTIONS

JE and SV developed the topic guide. JE and LY conducted the interviews and transcriptions. CA and EK performed coding and analysis, SV reviewed and approved themes. CA drafted the first version of the manuscript, and all authors reviewed and accepted the final version of the submitted paper.

### PEER REVIEW

The peer review history for this article is available at https://publons.com/publon/10.1111/jan.15055.

## Supporting information

Appendix S1

Appendix S2
