# Supplementary material for: Experiences of current vital signs monitoring practices and views of wearable monitoring: A qualitative study in patients and nurses
Source: J Adv Nurs. 2021 Oct 15;78(3):810–22. doi: 10.1111/jan.15055 (PMC9293408; doi:10.1111/jan.15055)
Supplement: Supplementary file 1 — Appendix S1 [file JAN-78-810-s001.docx]

# Appendix 1 – Interview topic guide

Table 2 – **Patient interview topic guide questions**.

| **Introduction to the topic:** “*During your admission, a bedside monitor may have continuously monitored you or you may have had nurse come and take your blood pressure, oxygen levels and temperature. Can you talk to me about the monitoring that you have experienced as a patient?*”. |
| --- |
| 1.          Was there anything good or bad about this? |
| 2.          Were you ever the only patient in your bay, who had a continuous monitor on? How did that feel? |
| 3.          Did you experience any alarms from these systems? Tell me a little bit about that? |
| 4.          Was your sleep disturbed in any way? And for what reasons? |
| 5.          In the context of your other invasive lines (drip or drain), how did you feel about the wired monitoring? |
| 6.          Was your independence affected as an in-patient? What was the reason for this? |
| 7.          Did you like being monitored? |
| 8.          Do you think anything could improve this part of healthcare? |
| **Introduction to ambulatory monitoring systems:** *“We’re trying to do is to develop monitors, so small wearable monitors that can pick up heart rate, oxygen levels, respiratory rate, how do you think that type of monitoring would be received by patients?”* |

Table 3 –**Nurses interview topic guide questions**.

| **Introduction to the topic:** “W*e are trying to understand the practice of monitoring in your clinical area, how it helps you to make the decisions that you do. “* |
| --- |
| 1. Can you describe how you identify a patient has deteriorated or clinically changed? |
| 1. Do you like to have the ability to continuously monitor your patients? |
| 1. What do you think that continuous monitoring adds to the clinical management of patients? |
| 1. How well do you think continuous monitoring is tolerated by your patients? |
| 1. Can you explain the process of escalation of care? |
| 1. What can make the process of escalation easier or harder? |
| **Introduction to ambulatory monitoring systems:** *“There are systems out there that mean that we can continuously monitor patients but they are a very small piece of kit, that means they can move around freely, but it means that you are continuously getting the data feed from this system.”* |
| 1. Is there anything, any important features that you can think of that you would want to see as a nurse, if you had one of these systems on your patient? |
