# Supplementary material for: Experiences of current vital signs monitoring practices and views of wearable monitoring: A qualitative study in patients and nurses
Source: J Adv Nurs. 2021 Oct 15;78(3):810–22. doi: 10.1111/jan.15055 (PMC9293408; doi:10.1111/jan.15055)
Supplement: Supplementary file 2 — Appendix S2 [file JAN-78-810-s002.docx]

# Appendix 2 – Coding Tree and theme development

## Theme 1- Vital sign data as evidence for escalation

### Clinical judgement

Clinical judgement

Clinical need for monitoring

Deterioration signs

Deterioration without triggering

Doctor seniority

Doctor’s experience

Nurse experience

Knowing the patient

Prioritisation

Severity of the situation

Close monitoring

### Patient escalation

Clinical escalation from alarms

Current escalation of care

Delayed escalation

Escalation without triggering

Doctor review with deterioration

Escalation at night

Escalation process

Getting doctors attention

ICU stay

Observation trends

Observations and clinical management

SEND

Sepsis

Track and trigger

Triggering with no deterioration

ICU support

Staff levels

## Theme 2- Trustworthiness of vital sign data

Manual observations

Performance of monitoring

Respiratory rate reliability

Technology

Reliability of continuous monitoring

Unexpected observation results

Reactions to abnormal observations

Baseline observations

## Theme 3- Finding a balance between continuous and intermittent monitoring

### Intermittent versus continuous monitoring

Continuous versus intermittent

Current monitoring practices

Locations

Mobilising

Monitoring invasiveness

Monitoring tolerance

Patient concern from observations

Predictability of monitoring

Thoughts on current practice

Independence

Tangled wires

Observations over night-time

### Human factors

Encouragement

Human factors

Patient safety

Relevance on monitoring

Staff frustration

Staff working relationships

Facilitating Data Entry

Reports of good care

SEND updates

### Practicalities

Benefits of continuous monitoring

Demographic relations to monitoring

Frequency of observations

Monitoring versus clinical equipment

Patient frustration

Save nursing time

Whiteboard

Continuous monitoring prompting other tasks

Thoughts on continuous monitoring

Poor patient compliance

Reassurance

Staff and patient interactions

### Noise pollution and alarm thresholds

Bleep systems

Alarms

Sleep disturbances

Staff reactions to alarms

Noise pollution was really crazy

Requesting help from alarms

Too unwell to notice alarms

## Theme 4- Ambulatory wearable devices for clinical monitoring

Ambulatory monitoring reliability

AMS alerts and EPR

AMS and alerting systems

AMS views

Benefits of ambulatory monitoring

Wearability of monitoring
